# Tuberculosis Heresies?

**Published:** 1920-10-30

**Authors:** 


### TUBERCULOSIS HERESIES?

TUBERCULOSIS is not only chief of the great diseases, it is more than correspondingly pre-eminent in medical literature. The latter distinction is not, however, very fruitful. The study of tuberculosis is like the game of lawn tennis—almost everyone has some knowledge of it, but very few a first-class knowledge. And since this study is now well on the way to constituting a specialty, one cannot expect such a state of affairs to change. Of course, thereby destructive criticism becomes easy, just as it is easy for the Wimbledon player to shake his head over garden-party performances. The point is, however, that the Wimbledon player would not take them seriously. Similarly, the medical profession should regard it as somewhat of a waste of time (not to mention paper and printers' labour) for rank errors in phthisiology to be seriously arraigned. Yet this, in the main, is what Dr. Marcus Paterson has done in a book * lately published. He begins by pointing out that butter, as well as milk, may contain tubercle bacilli. But did not Kanthack—who died ever so many years ago—as well as sundry foreigners, prove this very point? When he protests that a sanatorium superintendent has not "got a cushy job," he is plagiarised by another dead man, Dettweiler to wit. He maintains that a negative sputum analysis has no value: but who says it has? Or that the Von Pirquet test is only useful in children?—why, he should have gone further, and said only in quite young children. Surely the statistical investigations made long since at Brompton and Mount Vernon Consumption Hospitals make supererogatory his fifty-third chapter, devoted to demolishing the thesis that it is dangerous to visit a tuberculosis hospital. In more than half the book, then, the author is merely thrice slaying the slain.

These needed deductions made, there is a residue of some value. "That good housing will eradicate tuberculosis" is a proposition certainly in need of confutation. And the idea of the book is good, each chapter the pillorying of an heresy. But the discrimination wants to be finer, the detail to be carried much further, and, in truth, the erudition to be somewhat greater. Of course, such increased connotation would involve lessened extension: the book would appeal to a much smaller audience, if a choicer one. It comes to this, that we do not want our Dr. Paterson to write books—others can do that. When, if ever, our knowledge of the disease simplifies its administrative control to the dimensions of that of yellow fever, or of malaria, then he, or one of his great organising ability, will certainly do here what Surgeon-General Gorgas did in Panama. Unfortunately that day is not yet. Unfortunately, too, one cannot remember that Gorgas had reputation as investigator or writer. Dr. Paterson proved that consumptives could stand hard manual work; the mechanism of tuberculous auto-inoculation was examined by Wright, and the disciples Meakin, Wheeler, and Inman. Dr. Paterson's name will live as the best sanatorium superintendent his country has produced—and this second book of his now joins the first one in a fellowship not unlike that of the sculptor Chantrey's brace of woodcock. Who does not remember their—" . . . unhappy lot, a sportsman carved them, whom an artist shot"?

* *The Shibboleths of Tuberculosis*: John Murray, London. 1920. 10s. 6d. net.

